# Long-term retrospective observation reveals stabilities and variations of hantavirus infection in Hebei, China

**DOI:** 10.1186/s12879-019-4402-8

**Published:** 2019-09-02

**Authors:** Shiyou Liu, Yamei Wei, Xu Han, Yanan Cai, Zhanying Han, Yanbo Zhang, Yonggang Xu, Shunxiang Qi, Qi Li

**Affiliations:** Hebei Key Laboratory of Pathogens and Epidemiology of Infectious Diseases, Institute for Viral Disease Control and Prevention, Hebei Provincial Center for Disease Control and Prevention, 97 Huaian East Road, Shijiazhuang, 050021 Hebei China

**Keywords:** Hemorrhagic fever with renal syndrome, *Rattus norvegicus*, Hantavirus, Seoul virus (SEOV), Epidemiology

## Abstract

**Background:**

Hemorrhagic fever with renal syndrome (HFRS) is an emerging zoonotic infectious disease caused by hantaviruses which circulate worldwide. So far, it was still considered as one of serious public health problems in China. The present study aimed to reveal the stabilities and variations of hantavirus infection in Hebei province located in North China through a long-term retrospective observation.

**Methods:**

The epidemiological data of HFRS cases from all 11 cities of Hebei province since 1981 through 2016 were collected and descriptively analyzed. The rodent densities, species compositions and virus-carrying rates of different regions were collected from six separated rodent surveillance points which set up since 2007. The molecular diversity and phylogenetic relationship of hantaviruses circulating among rodents were analyzed based on partial viral glycoprotein gene.

**Results:**

HFRS cases have been reported every year in Hebei province, since the first local case was identified in 1981. The epidemic history can be artificially divided into three phases and a total of 55,507 HFRS cases with 374 deaths were reported during 1981–2016. The gender and occupational factors of susceptible population were invarible throughout, however age of that was gradually aging. The annual outbreak peak always present in spring, while the main epidemic region had gradully altered from south to northeast. Surveillance of rodents revealed that residential rodents significantly possessed higher density and virus-carring rate than field rodents. The house rat, *Rattus norvegicus*, was the dominant rodent species and *Seoul virus* S3 sub-genotype which is continued but slightly evolving perhaps to be the sole pathogen for local HFRS cases of Hebei province.

**Conclusions:**

This long-term province-wide surveillance and epidemiological analysis has revealed the stabilities and variations of hantavirus infection in North China. In order to improve current prevention and control strategies of HFRS in China, all surveillance should be continuously enhanced and variations should be paid more attentions.

**Electronic supplementary material:**

The online version of this article (10.1186/s12879-019-4402-8) contains supplementary material, which is available to authorized users.

## Background

Hemorrhagic fever with renal syndrome (HFRS) is an emerging zoonotic infectious disease caused by hantaviruses which circulate worldwide posing the ongoing public health threat in most countries of Europe and Asia [1–3]. HFRS emerged in China among the early 1930s, and to date 29 of 31 provinces had reported the epidemics of this zoonotic disease. So far, 60,000–100,000 HFRS cases are collectively reported worldwide each year, with the majority occurring in China [4, 5]. Thanks to lots of comprehensive preventive measures, such as the national immunization programs, a dramatical decline of the incidence of HFRS among China was observed since the beginning of the new century [4]. Moreover, sporadic cases of oriental-HFRS (induced by hantavirus of Asian serotype) were also irregularly reported in the Americas and Europe over the years [6, 7]. However, it has been suggested that the real epidemic occurring in Western World were probably underestimated or even neglected because of the lower medical awareness for detecting milder or atypical cases [8–10]. Human infections with hantaviruses are generally associated with inhalating the aerosolized excreta of virus-carring rodents [2].

Hantaviruses now have been considered as a single family, *Hantaviridae* belonging to the *Bunyavirales* order [11]. So far, more than 50 hantavirus species divided into three phylogenetic clusters have been identifed from rodents, shrews, moles and bats, but only rodent-borne hantaviruses have been found to cause pathogenicity in humans [1, 6]. Due to the millions of years co-evolution with reservoir hosts, different hantaviruses possess various host specificities, geographical tropisms and pathologies in humans [1, 2]. It has been identified that HFRS in Asia is primarily caused by either *Hantaan virus* (HTNV) or *Seoul virus* (SEOV). HTNV usually causes more severe symptoms than SEOV and they were naturally carried by *Apodemus agrarius* and *Rattus norvegicus*, respectively [6]. However, it should be concerned that anthropogenic changes in host reservoir ecology and biodiversity have enhanced the risk of virus reassortment and host spill-over [2, 12].

As a member from *Bunyavirales*, hantavirus possesses a tri-segmented and negative single-stranded RNA genome consisting of small (S), media (M) and large (L) segment, which encodes nucleocapsid (N) protein, glycoprotein precursor (GPC) and RNA-dependent RNA polymerase (RdRp), respectively. During the virus replication, GPC will be co-translationally cleaved into the glycoproteins Gn and Gc by cellular signal peptidase. The heterodimer of Gn and Gc will be finally constructed into spikes and appeared upon the virion surface [1]. It had been suggested that the viral envelop glycoproteins play an important role in recognizing target cell, interacting with entry receptors, and stimulating host immune response [1, 2]. Meanwhile, GPC gene was considered as common genetic variable site in different hataviruses making it ideal segment for genotyping [13].

**Fig. 1:**
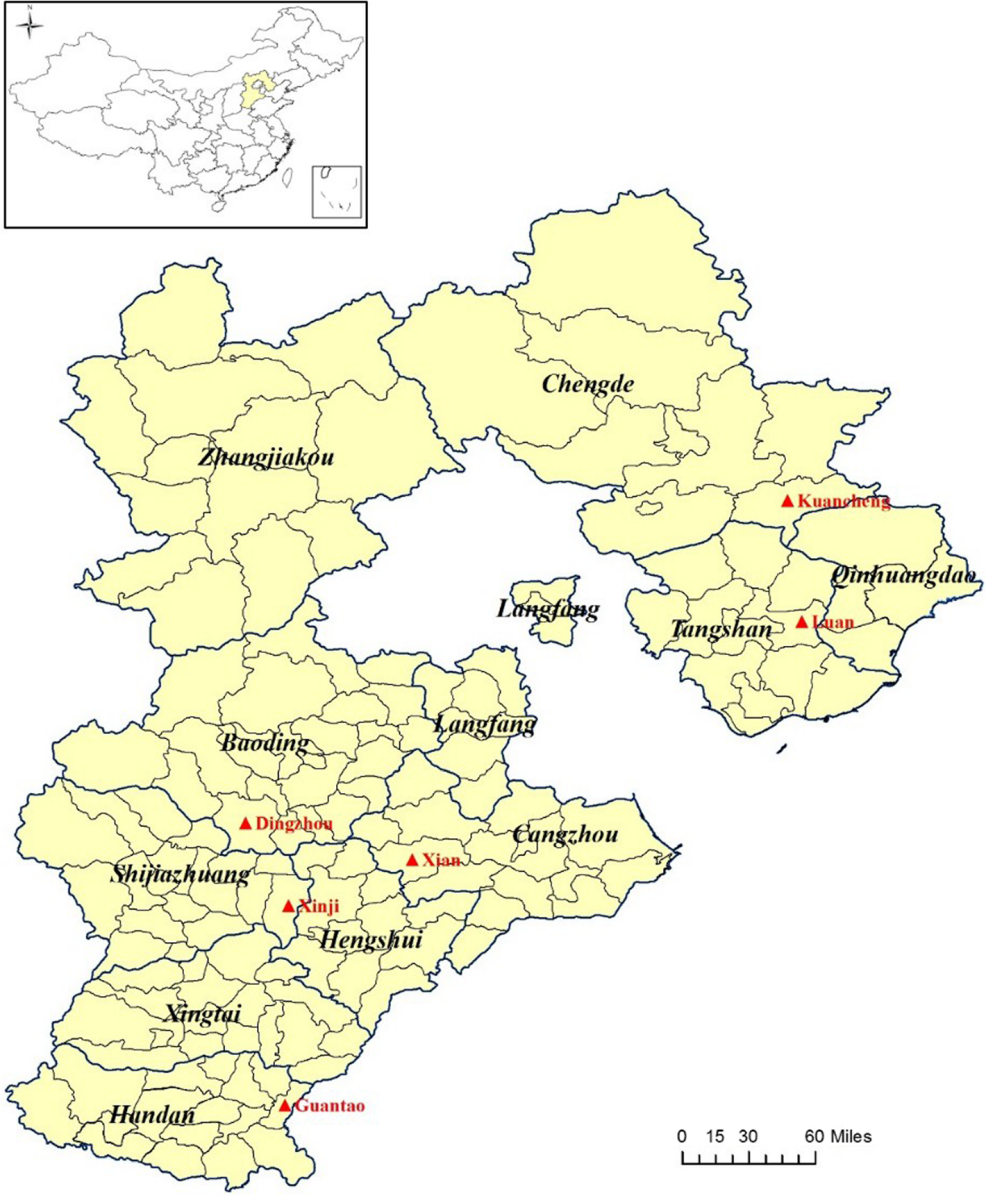
Map of Hebei province with location of rodent surveillance points (▲). Special acknowledgement to DataV.GeoAtlas (Alibaba, China) for the source of vector map

The province of Hebei is located in the Northern China plain, and surrounds Beijing, the capital of China (Fig. 1). In this study, we collected and analyzed the 36 years epidemiological data of HFRS cases and hantavirus infection in rodents of Hebei province. The long-term surveillance has revealed that hantavirus infections exhibited both stable and variational properties in this endemic area. The results help us comprehensively understand the epidemiology and etiology of HFRS infection and warned us surveillance of hantaviruses infected in rodents was helpful and necessary for HFRS control and prevention.

## Methods

### Collection of data for HFRS cases

The course of HFRS can be ranging from frequently asymptomatic to a lethal outcome [1], and here we have only collected epidemiological data of cases that with typical HFRS symptoms. Cases of HFRS, as one of category B notifiable infectious diseases in China, have been reported to the National Notifiable Infectious Diseases Reporting Information System (NIDRIS) in real time (within 12 h). NIDRIS was implemented since 2004 and has covered all health care institutions across China [14, 15]. Epidemiological data of HFRS patients used in this study were mainly collected from NIDRIS and data before 2004 were supplementally retrospected from our manual records. Before 1982, HFRS cases were only defined by a national standard of clinical criteria. Since 1982, cases were further confirmed by detecting specific antibodies (IgM and IgG) against hantavirus antigens in serum samples.

### Collection of rodent specimens

In order to figure out the potential rodents population and virus-carrying rate of Hebei province, four separated surveillance points (Luan county, Xinji city, Dingzhou city and Xian county) were set up since 2007 and two more points (Guantao county and Kuancheng Man Autonomous county) were added since 2015 (Fig. 1). In each surveillacnce point, the rodents were captured with rattraps and baited with peanuts according to related National Survellance Program during annual spring and autumn. In field areas, which include grassland, woodland and footpath besides farms, the rattraps were set as parallel lines letting interval distance as 20 m for each line and 5 m for each rattrap. Set one rattrap per 10–20 square meters in residential areas, including granary, barn and outdoor cooking bench, where had been obtained individual consent from local residents. All rattraps were set before night and recovered in next morning. After identification of species through morphological observation, captured rats were narcotized by pentobarbital sodium, then dissected and extracted lung tissue sterilely. Lung tissues of rodents were immediately stored into liquid nitrogen until further laboratory test. All sections of this report adhere to the ARRIVE Guidelines for reporting animal research. A completed ARRIVE guidelines checklist is included in Additional file 1.

### Immunofluorescent assay (IFA), reverse transcription (RT)-PCR and sequencing

Lung tissues of rodents were conducted into frozen sections to detect hantavirus antigen by IFA with rabbit anti-SEOV/L99 and HTNV/76–118 hantavirus antibodies and FITC-labeled IgG according to the previous method [16, 17]. Since 2012, total RNA were extracted from lung tissues of rodents that had been confirmed for virus-carrying positive by IFA using TRIzol reagent (Invitrogen Life Technologies, Carlsbad, CA, USA) according to the manufacturer’s instruction. Then, cDNAs were synthesized with AMV reverse transcriptase (Promega, Beijing, China) in the presence of primer P14 which was genus-specific segment for hantavirus [18]. The partial M segment (1934–2353 bp, total 420 bp) were amplified by using pairs of primers and relative protocols discribed previously [17, 19], that now have been included into Chinese National HFRS Surveillance Strategy. After examination with agarose gel electrophoresis, RT-PCR products were purified and cloned into plasmid vectors as previously described [13]. The positive recombinant plasmids were send to Sangon Biotech (Shanghai) Corporation Ltd. for sequencing, and all processed sequences had been stored into GenBank (MK340822-MK340857).

### Phylogenetic analysis

All 36 nucleotides sequences that we obtained conbining some other M sequences of typical *Seoul virus* that have been stored in GenBank were aligned with ClustalX [20], trimmed off terminal un-aligned regions using Gblocks [21], and constructed into a neighbor-joining tree using MEGA 7 [22] with the maximum composite likelihood mode for distance calculation and 1000 replications for bootstrapping. The partial M segment of a strain of *Hantaan virus* was used to root the tree. Amino acids sequences of 36 *Seoul virus* were also aligned using same method mentioned above to figure out the variations with significance and the strain isolated from Qinhuangdao City, Hebei province in 1993 was used as a reference.

## Results

### Morbidity and case-fatality rate

**Fig. 2:**
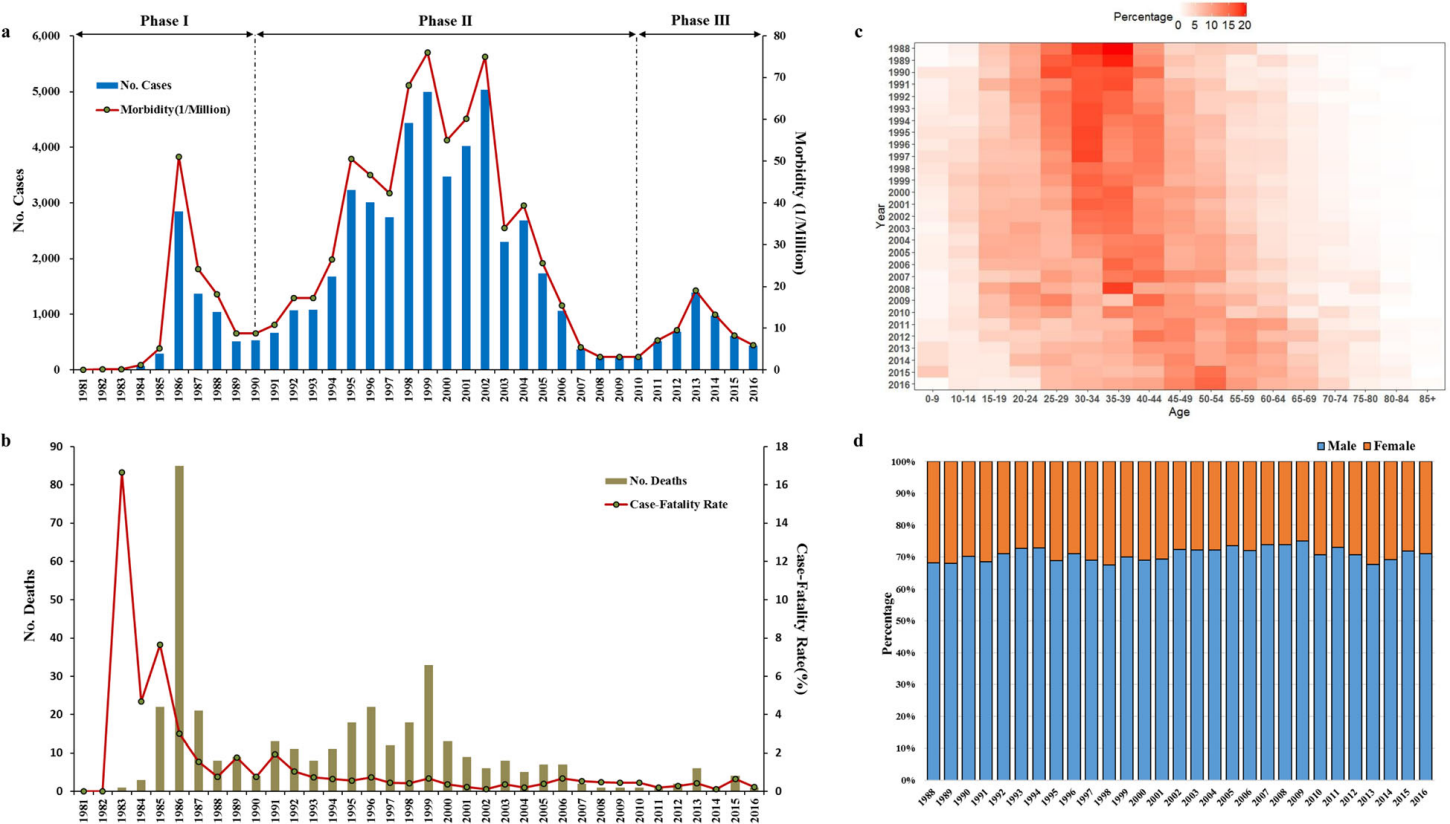
Morbidity, case-fatality rate and population distribution of HFRS cases in Hebei province. **a** Annual numbers and morbidity (case per million population) of HFRS patients reported since 1981 through 2016, which can be artificially divided into three phases according to the mobidity. **b** Annual numbers of deaths and case-fatality rates caused by HFRS since 1981 through 2016. **c** Annual age distributions of HFRS cases since 1988 through 2016. **d** Annual gender distributions of HFRS cases since 1988 through 2016

The first clinical human infection case of HFRS in Hebei province [23] was reported in 1981, and then the HFRS cases were continuously emerged every year. According to the morbidity, the history of hantavirus infections in human beings in Hebei province can be generally divided into three phases, 1981–1990 (Phase I), 1991–2010 (Phase II) and 2011–2016 (Phase III), within the 36-year period (Fig. 2a). In total, 55,507 HFRS cases with 374 deaths were reported in Hebei province and they almost involved all the area of the province. The largest province-wide HFRS outbreak occurred in 2002, which associated with 5037 cases, 6 deaths and more than 140 counties. The annual incidence of HFRS disease was instable, spanning from 0.02 through 76.00 cases per million population. Thanks to lots of comprehensive preventive measures conducted recently, incidences of the latest outbreak epidemic have been controlled under 20 cases per million population (Fig. 2a). At the earlier stage of emergency, the annual case-fatality rate of HFRS in Hebei province had reached up to 16.7% (1983), then it has been controlled at a relative lower level, spanning from 0.1–1.9% with an average as 0.6% (1987–2016) (Fig. 2b).

### Population distribution

People in any age group, from infants to elderly people, are considered to be generally susceptible for HFRS infection. While, the middle aged people (20 to 59 years old) accounted for the most (84.8%) cases. In addition, long-term surveillance also showed that annual age-peaks of HFRS patients were mildly but continuously aging since 1988 when age information of cases start to be collected (Fig. 2c). Expectantly, the gender of more susceptible population was invarible throughout that male patients with higher incidence always accounted for approximate 70% of the total number of HFRS cases (Fig. 2d). Farmer still was the most dominant occupational factor (data not shown), which was also consistant with that of other areas in China [6].

### Temporal and spatial distribution

**Fig. 3:**
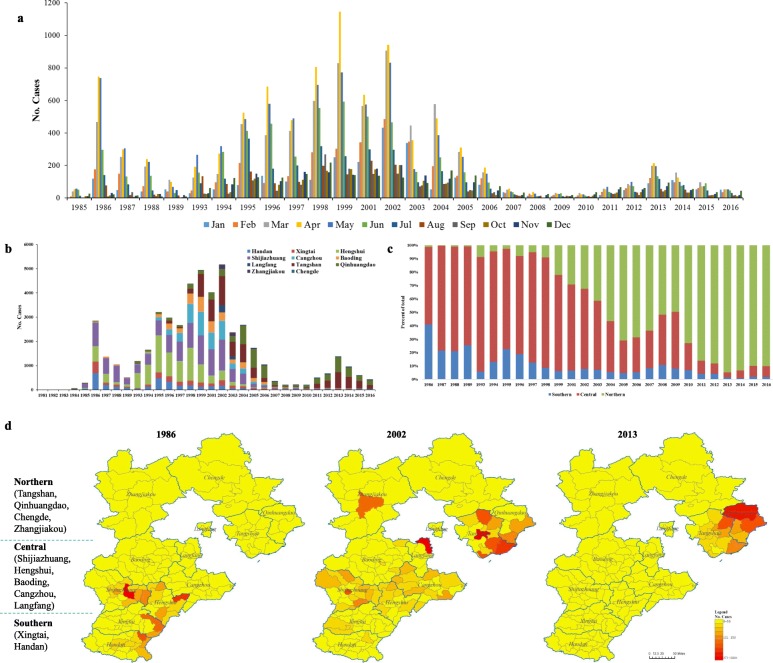
Temporal and spatial epidemiology of HFRS in Hebei province. **a** Monthly cases of HFRS patients reported from 1985 through 2016, except 1990–1992 and 2000 that were not recorded. **b** Annual numbers of HFRS patients reported from different cities (represent with different colors) since 1981 through 2016, except 1990–1992 and 2000 that were not recorded. **c** Annual percentages of HFRS cases reported from three regions (southern, central and northern reagion), since 1986 through 2016 except 1990–1992 and 2000 that were not recorded. **d** Geographic distribution of annual cases of HFRS reported in 1986, 2002 and 2013 representing three epidemic phase, respectively. Three regions of Hebei province were artificially separated. Special acknowledgement to DataV.GeoAtlas (Alibaba, China) for the source of vector map

Generally, HFRS outbreak emerged throughout the whole year, and there were two seasonal peaks, a bigger one in spring and a smaller one in winter [4]. The monthly surveillance of HFRS cases of Hebei province showed that the bigger peak and smaller peak appeared alternately during 1981–2010 (Phase I and II), however the winter smaller peak were gradually going to be unapparent since 2011 (Phase III) (Fig. 3a).

All 11 cities of Hebei province have ever been reported the HFRS cases, while the main outbreak areas shift gradually (Fig. 3b). If we divided Hebei province into three part (southern, central and northern area), the long-term surveillance revealed that the high incidence area had altered from southern and central area to northern area, especially in the northeast of the province (Fig. 3c, d). During the Phase I HFRS epidemic, most cases were reported from the cities in sourthern and central area and fewer cases were reported from the northern cities. Since Phase II, the proportion that cases from northern cities of provincial total incidences increased progressively. So far, cases from Tangshan and Qinhuangdao, which located in the northeast of the province, had accounted for nearly 90% of the total incidences.

### Rodent density, species composition and virus-carrying rate

**Fig. 4:**
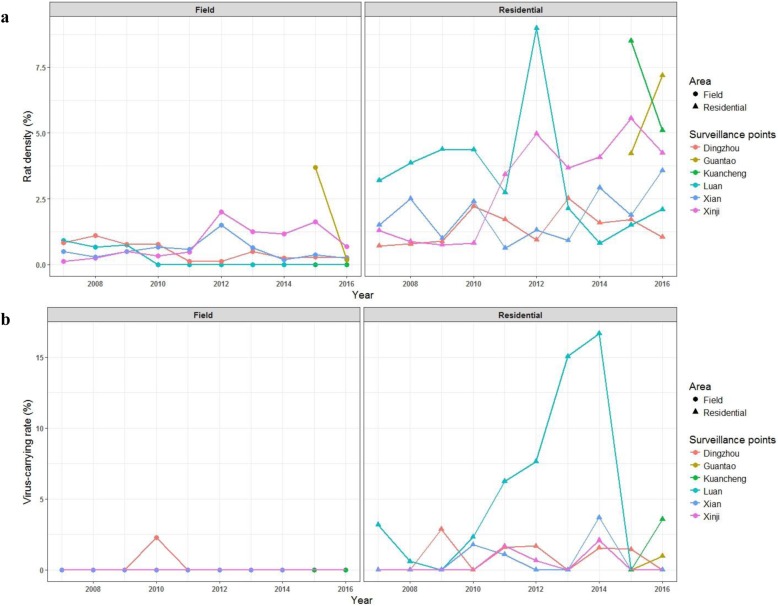
Annual surveillence of rodents population, including diversities (**a**) and virus-carrying rates (**b**)

Since 2007, in total of 181,966 rattraps were set up with 2721 rodents, including *Rattus norvegicus*, *Mus musculus*, *Tscherskia triton*, *Apodemus agrarius* and some other unrecognized species, were captured from the six surveillance points (Table 1, Additional file 2). While the annual rodent densities in each surveillance points had a great fluctuation, the residential area generally possess much higher rodent density than the field (U = 147.5, *P* < 0.001) (Fig. 4a). For the rodent species composition, no matter in residential area or the field *R. norvegicus* indisputably was the dominant rodent species and it accounted for 85 and 63% of all captured rodents, respectively. In addition, *M. musculus* was the second largest rodent group inhabitting in the residential area. *T. triton* and *A. agrarius* were only discovered in the field.

**Table 1:** The summary of rodent density, species composition and virus-carrying rate within six surveillance points since 2007 to 2016^a^

Area	No. rattraps	No. rats	Density	Virus-Carrying Rate	*Rattus norvegicus*	*Mus musculus*	*Tscherskia triton*	*Apodemus agrarius*	Other species
Field	89,367	471	0.53%	1.74%	298 (63%)	29 (6%)	127 (27%)	3 (1%)	14 (3%)
Residential	92,599	2250	2.43%	0.05%	1902 (85%)	342 (15%)	0	0	6

^a^The number of rattraps, rats and the individual species were the summation of all surveillance points. Density and virus-carrying rate were the average of all serveillance points. The percentages in brakets represent the proportions of the induvidual species to the all rodents captured in field or residential area

The lung tissues of all captured rodents had been detected of the hantavirus antigen by IFA. The result showed that the virus-carrying rates of field rodents were much lower than that of rodents living in the residential area (U = 551.0, *P* < 0.001) (Table 1, Additional file 2, Fig. 4b). All field rodents were detected as hantavirus free only except the rodents captured from Dingzhou City in 2010. The residential rodents of most surveillance points stably possess relative low virus-carrying rates (under 5%), while the virus-carrying rate of rodents captured from Luan county located in northeast of the province had kept a high level during the third epidemic.

### The molecular evolution of the virus

**Fig. 5:**
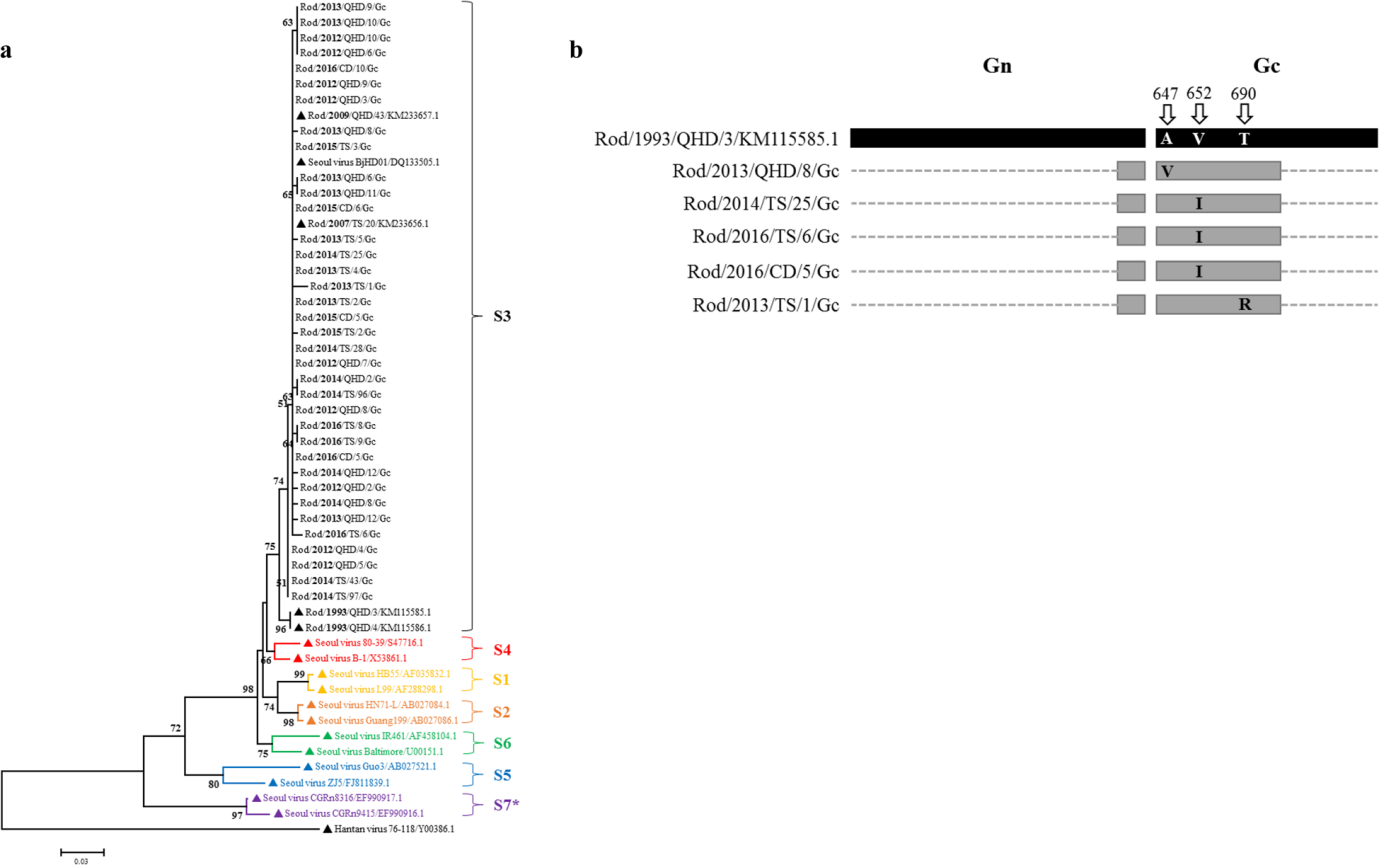
Phylogenetic analysis of partial M segments of hantaviruses circulating in Hebei province. **a** Phylogenetic tree of 36 SEOV identified from Hebei and other typical SEOV from different sub-genotypes (S1-S7, *S7 was considered as a result of natural reassortment between HTNV and SEOV [9]), based on partial M nucleotides sequences (1934–2353 bp, total 420 bp). A strain of HTNV was used to root the tree. Sequences marked with triangle (▲) were all extracted from GenBank. **b** Three sites of mutation in amino acids sequences identified in 5 of 36 SEOV. The strain isolated from Qinhuangdao city, Hebei province in 1993 was used as a reference

Not all virus-carrying lung samples detected by IFA for positive could be successfully detected by RT-PCR due to the different thresholds. Totally, only 36 lung tissues of rodents captured from the northeastern cities of the province, Chengde, Tangshan and Qinhuangdao where were the current high-incidence area for HFRS, were detected for positive by RT-PCR of partial M segment (1934–2353 bp, total 420 bp). The phylogenetic analysis of the nucleotide sequences showed that hantaviruses circulating in Hebei province evolved slowly and they all belonged to *Seoul virus* S3 genotype (Fig. 5a). It is shown that the hantaviruses identified in 1993 had an apparent genetic difference with the viruses identified in last decade. However, there was no significant genetic divergence between each hantaviruses identified during this ten-year period (2007–2016), neither in different areas nor different years.

All variations of the viral nucleotides we identified are substitutions without any indel and most of them are non-signification. Among the 36 amino acid sequences of the viral partial glycoproteins, three sites (A647V, V652I and T690R) of five sequences are shown with single mutation compared to the strain isolated from Qinhuangdao in 1993 (Fig. 5b). The partial glycoprotein gene we amplified covers the end of Gn gene and the head of Gc gene, and all three mutated sites are in this extra-membrane part of Gc protein that will be present on the surface of mature viral particle.

## Discussion

This province-wide long-term surveillance in northern China elucidates that the circulation of HFRS performs certain periodicity and now we are in the third epidemic season. The most awful period is the second epidemic which occurred for 20 years and effected almost all the cities of Hebei province. Thus, more attention need to be paid cause this zoonotic infectious disease has killed too many people and made severe adverse effects to humans [6]. Thanks to lots of comprehensive preventive measures, such as the national immunization programs, health promotion, environment improvement and deratization, the annual incidence of HFRS cases in Hebei province have been controled under 20 cases per million population in last decade. However the periodical recurrence of HFRS epidemic has warned us the surveillance of hantavirus infection in humans and rodents should be continuely enhanced.

For the first time, we have detected that the age peak of HFRS patients is slightly getting older. It is assumed that more and more young people trend to migrate into urban cities resulting in low frequency of contact with virus-carrying rodents. While, adult male farmers are still the key population of HFRS disease promptting that these people should be primary objects for the immunization programs. So far, the inactivated vaccines yielded from cultured cells or rodent brain has been licensed for use in humans in China [24] and the immunization program was executed in northeastern cities, Tangshan and Qinhuangdao since 2008. While the immune protective efficiency of these vaccines was still unclear and needs some further observation. In addition, some other classical or advanced molecular vaccine approaches, including DNA vaccines and attenuated live vaccines are still in pre- or clinical stages for further evaluations [6, 24].

The bigger spring peaks of HFRS were obvious and consistent annually warning people to decrease the frequency of contaction with rodent populations at this time. While, it is hard to interpret that the smaller winter peaks are going to be unapparent. Previous epidemiological study has elucidated that the winter peak was generally associated with HTNV carried by striped field rat (*A. agrarius*) and the spring peak was mainly caused by SEOV carried by house rat (*R. norvegicus*) [25, 26]. The decline of winter peak potentially interprets the proportion of HFRS patients associated with HTNV is decreasing, which coincides with pathogenical surveillance of rodents that only SEOV were identified in Hebei province during latest decade. On the other hand, the previous apparent winter peaks especially during second epidemic demonstrated that HTNV might circulate in Hebei province once.

Based on this long-term surveillance, we discovered that the high incidence region of HFRS has gradully shift. While, the principal factor of HFRS transmission dynamic is still unclear. It has been suggested that the climate variability may drive the dynamics of human HFRS infection, because the climate, such as temperature and rainfall can provide favorable conditions for both rodent population growth and virus transmission [27–29]. Urbanization and other relative human activities may also affect the frequency of contact between human and rodent populations furtherly impacting HFRS transmission dynamic [30, 31]. However, it seems to be unconvincing to consider urbanization playing key role in high incidence region shift cause urbanization of central or southern part of the province was comparable to northeastern part. In addition, it has been proven that there are some potential correlationship between incidence of HFRS and air pollution [32]. Herein, we give a hypothesis that since 1980s the ecological environmental factors and human activities made a combined effection to local host species and virus genotype furtherly deciding the high incidence region shift.

As a zoonotic disease, the incidence of HFRS has a close relationship with the local population of rodents, including host density and virus-carrying rate [33]. The annual host densities in Hebei province were drastically fluctuated indicating that the rodent habitted ecology environment was flimsy. While an apparent peak of virus-carrying rate was solely observed in Luan county, where was the high-incidence region of HFRS during the third epidemic phase indicating that virus-carrying rate play a more significant role to affect incidence of HFRS infection. Therefore, virus-carrying rate should be considered as a significant surveillance indicator to alert next HFRS epidemic. In addition, deratization especially in the residential area should be encouraged, cause the higher host density has made people under high exposure risk of rodent-borne pathogenic viruses including hantaviruses.

Epidemiological characteristic and clinical manifestation of HFRS can be determined by virus genotypes which are further influenced by host speicies [1]. In central China, HFRS has stronger relationship with HTNV carried by striped field mouse [27]. It has been shown that HTNV and SEOV simutaneously circulated in most areas of China, such as eastern coastal city, Qingdao [33], central city beside Yangzi River, Wuhan [34] as well as southwestern area [9] and northeastern area [35]. Uniquely, we have never identified HTNV among rodents and correspondingly the field rat, *A. agrarius* the natural host for HTNV, was also seldom to be found even in the field areas of Hebei province since 2007. However, the house rat, *R. norvegicus* predominantly existed in both residential areas and field areas leading SEOV probably to be sole pathogen for HFRS infection in this northern China province. For the past few years, more and more evidences has proven that due to global warming or the usage of rodenticides the asian house rat, *Rattus tanezumi* which naturally exit in southern China has expanded their vivosphere northward and aggressively influenced local rodent population including *R. norvegicus* [36, 37]. Because so far *R. tanezumi* has not been proven to be a natural host for SEOV, this migration might incompletely explain the shift of HFRS high incidence region of Hebei province. Thus far, in order to comprehensively illustrate the transmission dynamic of HFRS, continuous surveillance of rodent population should be enhanced in the future.

Based on phylogenetic analysis, we have known that hantaviruses circulated among high-incidence region of HFRS evolved slightly during the past decade and there was no apparent geographic segregation indentified. Here we modestly gave a hypothesis that there was no extraneous hantavirus invaded recently and only native strains of hantavirus were circulated throughout in Hebei province from 1990s to date. Meanwhile it has been shown that SEOV, probably the only hantavirus species which globally spread, has spread into west world owing to the omnipresence of its rodent host [38, 39]. The only valid explanation that why SEOV-HFRS epidemic situation of China was much more serious than that of western countries over past decades, is and remains a lower medical awareness in the West for detecting milder and sometimes atypical SEOV-HFRS cases, which are probably underreported [10]. Most mutations of nucleotides of SEOV identified in this study were considered to be no significance and two sites with subtitutions of amino acids in separated strains were supposed to occur occasionally. However the amino acid mutation (V652I) simutaneously present in three strains should be gaven more attention. It has been proven that both Gn and Gc are transmembrane proteins playing significant roles in recognition, adhesion and invasion of target cells [2]. Thus, further surveillance and laboratory study should be done to figure out whether this mutation affects physiology and transmission dynamic of viruses and clinical manifestation of HFRS.

## Conclusions

In conclusion, this long-term province-wide surveillance in northern China indicates that HFRS epidemic periodically occurred and the high-incidence region has shift. SEOV carried by house rat *R. norvegicus* probably is sole pathogen for HFRS in Hebei province and further molecular evidence elucidates that viruses circulated during past decade are homologous but they are still slightly evolving. Continued surveillance of hantavirus infection in both humans and host rodents is needed to fight against this infectious zoonotic disease.

## Additional files

Additional file 1: A completed ARRIVE guidelines checklist. (PDF 1208 kb)

Additional file 2: Complete statistics of rodent density, virus-carrying rate and species composition of each surveillance points in Hebei province since 2007 to 2016. (XLSX 15 kb)

## Data Availability

The datasets supporting the conclusions of this article are included within the article and the additional files. The original data of this article are held at Hebei Center for Disease Control and Prevention, and data can be accessed from the corresponding author upon reasonable request. Due to patient confidentiality some of the data cannot be accessed.

## Abbreviations

HFRS: Hemorrhagic fever with renal syndrome
HTNV: *Hantaan virus*
IFA: Immunofluorescent assay
NIDRIS: National Notifiable Infectious Diseases Reporting Information System
RT-PCR: Reverse transcription-polymerase chain reaction
SEOV: *Seoul virus*
